# The effect of COVID-19 on nuclear medicine and radiopharmacy activities: A global survey

**DOI:** 10.1038/s41598-023-36925-4

**Published:** 2023-06-28

**Authors:** Fatma Al-Saeedi, Peramaiyan Rajendran, Dnyanesh Tipre, Hassan Aladwani, Salem Alenezi, Maryam Alqabandi, Abdullah Alkhamis, Abdulmohsen Redha, Ahmed Mohammad, Fahad Ahmad, Yaaqoup Abdulnabi, Altaf Alfadhly, Danah Alrasheedi

**Affiliations:** 1 The Department of Nuclear Medicine, College of Medicine, Kuwait University, P.O. box: 24923, 13110 Safat, Kuwait; 2 Department of Biological Sciences, College of Science, King Faisal University, AlAhsa, Saudi Arabia; 3 Department of Biochemistry, Saveetha Dental College & Hospitals, Saveetha Institute of Medical and Technical Sciences, Chennai, 600 077 Tamil Nadu India; 4 Translational Biomedical Imaging Laboratory, Department of Radiology, Children’s Hospital Los Angeles, Los Angeles, California USA; 5 College of Medicine, Kuwait University, P.O. box: 24923, 13110 Safat, Kuwait

**Keywords:** Health care, Medical research

## Abstract

Globally, COVID-19 affected radiopharmaceutical laboratories. This study sought to determine the economic, service, and research impacts of COVID-19 on radiopharmacy. This online survey was conducted with the participation of employees from nuclear medicine and radiopharmaceutical companies. The socioeconomic status of the individuals was collected. The study was participated by 145 medical professionals from 25 different countries. From this work, it is evident that 2-deoxy-2-[18F]fluoro-D-glucose (2-[^18^F]FDG), and ^*99m*^*Tc-labeled* macro aggregated albumin ^99m^Tc-MAA were necessary radiopharmaceuticals used by 57% (83/145and 34% (49/145;) respondents, respectively for determining how COVID infections affect a patient’s body. The normal scheduling procedure for the radiopharmacy laboratory was reduced by more than half (65%; 94/145). In COVID-19, 70% (102/145) of respondents followed the regulations established by the local departments. Throughout the pandemic, there was a 97% (141/145) decrease in all staffing recruitment efforts. The field of nuclear medicine research, as well as the radiopharmaceutical industry, were both adversely affected by COVID-19.

## Introduction

COVID-19 is an acute infectious disease since the “Spanish” influenza pandemic that occurred in 1918. The current COVID-19 pandemic is considered to be one of the most massive and chaotic challenges that are currently confronting global public health^1–5^. On a global scale, the COVID-19 outbreak has resulted in a significant number of deaths and economic losses, it affected life and lifestyle. Furthermore, the outbreak has caused uncertainty and it has posed a risk to the medical sectors and relevant persons^6–10^. Despite this, a survey that can provide a more detailed explanation of the pandemic, report on its effects, and forecast its potential future effects is needed, as very limited research reports are published in this domain. The current COVID-19 outbreak has introduced scientists to a pandemic challenge that necessitates optimal levels of information exchange about the current outbreak as well as any future outbreaks. This information exchange must take place outside of the limitations imposed by physical geography, climatology, and scientific inquiry. COVID-19 is a worldwide infection that can affect a wide range of medical professionals, also confronting the duties, activities, and work performed in radiopharmacy laboratories.

We were interested in determining how we could assess the impact of COVID-19 on the operations and services provided by the department of nuclear medicine, specifically the radiopharmacy lab, which is the department’s central division. The radiopharmacy laboratory is the site where many radiopharmaceuticals are produced, where their quality is monitored, and even where they are stocked in inventory. Our study aimed to develop a preliminary database for our international survey and to assess the impact of the COVID-19 pandemic on the research and commercial interactions of radiopharmaceutical companies.

## Methods

### Study design

This survey was conducted between the 15th of September 2021 and the 10th of October 2022 with nuclear medicine and radiopharmaceutical staff members from all over Kuwait and other countries. The survey was conducted after obtaining approval for the survey’s protocol (No. 1762/2021) from Kuwait’s ethics committee as well as the Ministry of Health-Kuwait. We confirm that all methods were performed in accordance with the relevant guidelines and regulations. We have collected the consent forms that were filled out by the participants, and they also signed it. Local governmental health areas in Kuwait, Saudi Arabia, the United Arab Emirates, Iraq, Jordan, Sudan, Morocco, Pakistan, India, the United States of America, the United Kingdom, France, Spain, Germany, Greece, Italy, Sweden, Brazil, Bosnia and Herzegovina, Turkey, South Africa, Tanzania, Colombia, Mexico, and Peru were represented by online survey respondents. The questionnaire was conducted in English. To achieve our objectives, we devised the simple idea of conducting a survey of the local residents. We searched for any fundamental information in different databases that could help us get started. We were astounded to discover so few studies on nuclear medicine procedures, particularly in radiopharmacy laboratories. The survey sought to learn more about COVID-19’s impact on the radiopharmacy industry and its activities.

### Sampling

During the pandemic, a web-based (online) questionnaire built on the Google platform was developed to investigate and collect data on the uses and activities involving radiopharmacy. The Centre for Research Support and Conferences of the University’s Faculty of Medicine and Health Sciences facilitated the production and distribution of email announcements. The participants were also provided with a link to a Google Form-generated web-based survey.

### Data collection

The semi-structured questionnaire was divided into two parts: the first, which consisted of five questions, asked for demographic information, and the second, which inquired about respondents’ levels of knowledge, attitude, activities, and practises regarding radiopharmacy during a pandemic. The questionnaire was made up entirely of closed-ended questions that required a response in the form of one or more checkboxes to indicate the desired level of response. The questionnaire was designed in such a way that each individual participant had seen the same set of questions in the same order. Each question was intended to be succinct and to the point. To increase the dependability of the responses, they were all recorded using the identical method. Each questionnaire form was subjected to a series of repeated checks to determine its content's reliability. The link to Google Forms and Microsoft Teams was posted, distributed, and circulated via a variety of social media platforms.

### Statistical analysis

We used descriptive statistics to characterise the population, their purchase preferences, and their scheduling activities. The paired sample *t* test was used to determine the reduction in the purchase of materials and radiopharmaceuticals in comparison to normal purchase status before the COVID-19 pandemic. A p-value of 0.05 was statistically significant.

## Results

### The socio-demographic profile of the participants

**Figure 1:**
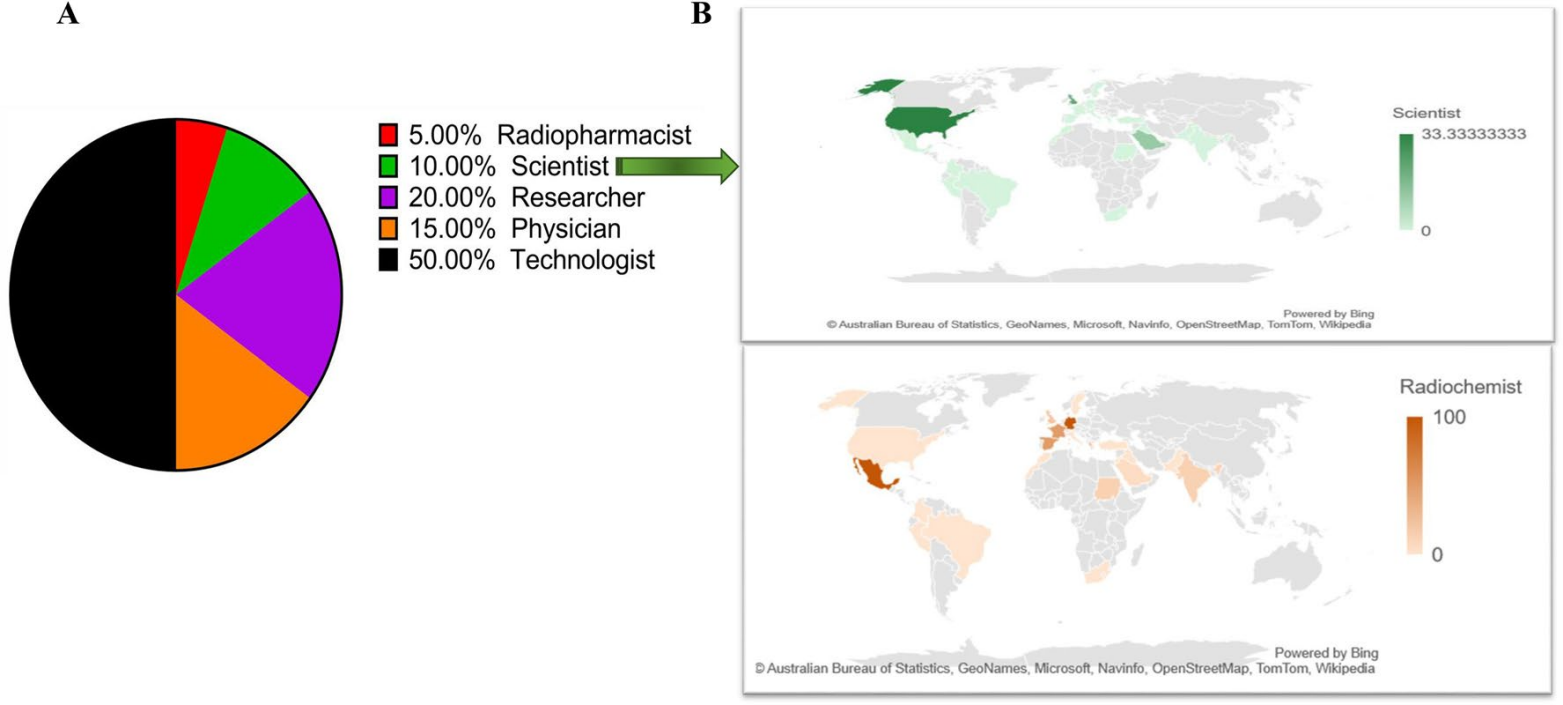
The socio-demographic pattern of participants surveyed. (**A**) profession (**B**) profession examples (scientist and radiochemist) among participants around the world.

Participants in the survey were classified based on where they worked, where they were from, what occupation they held (Fig. 1), and age. During the duration of the study, 145 medical professionals from 25 different countries participated and completed the questionnaire. The participants who had completed the survey included 20% (29/145) researchers, 15% (22/145) physicians, 10% (15/145) scientists, 2% (3/145) radiopharmacists, 0.7% (1/145) radiochemists, 0.7% (1/145) radiopharmaceutical chemists, 1.37% (2/145) physicists, 1.37% (2/145) cyclotron operators, and technologists who accounted for the remaining of 50% (73/145) of the survey responses.

Sixty percent (87/145) participants worked in radiopharmacy; and among them 26% (23/87) worked in PET centres; and 26% (23/87) worked in research. The majority of them were employed in the radiopharmacy section of the Nuclear Medicine Department.

**Figure 2:**
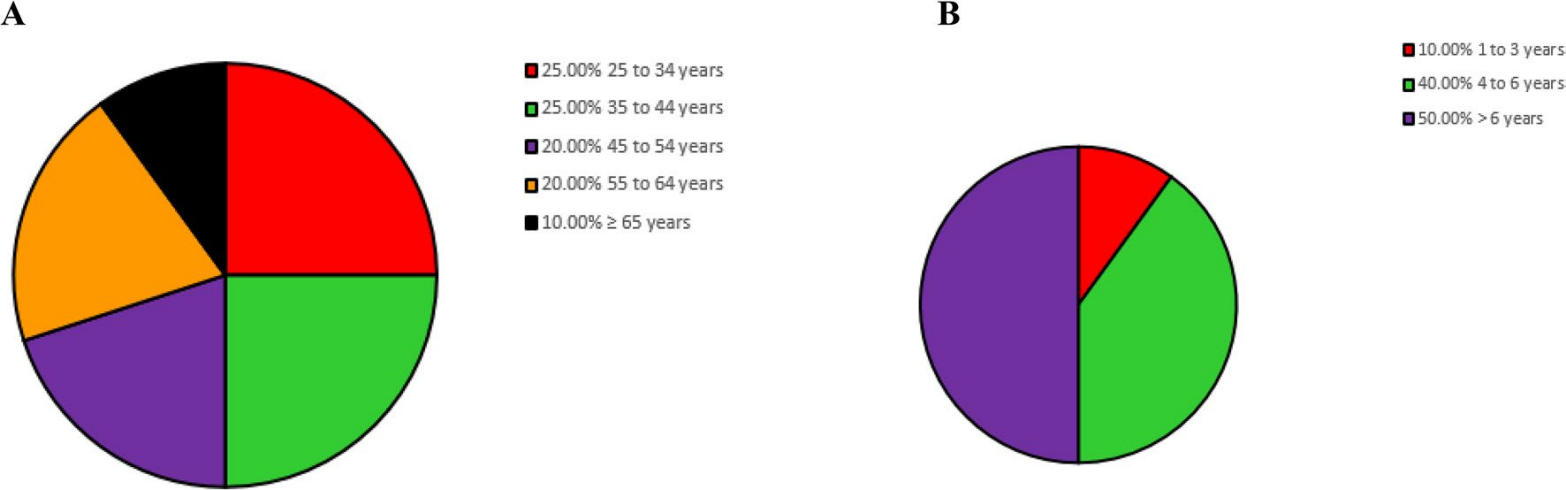
The socio-demographic pattern of participants surveyed. (**A**) age (**B**) work experience.

The age distribution of the respondents shows that 25% (36/145) fall into the age range of 25–34 years and 25% (36/145) workers fall in the range of 35–44 years. The percentage of people who are employed is comparable across both age groups. The remaining 50% percent comprised of people aged 45 to 54 years (20%, 29/145, 55 to 64 years (20%, 29/145), and 65 years and above (10%; 15/145) as shown in Fig. 2A. According to Fig. 2B, the majority of the participants (50%; 73/145) had more than 6 years of experience, followed by 4 to 6 years (40%; 58/145) and 1 to 3 years (10%; 15/145).

### The radiopharmacy activities and the measurements

#### Purchase and preparation

**Figure 3:**
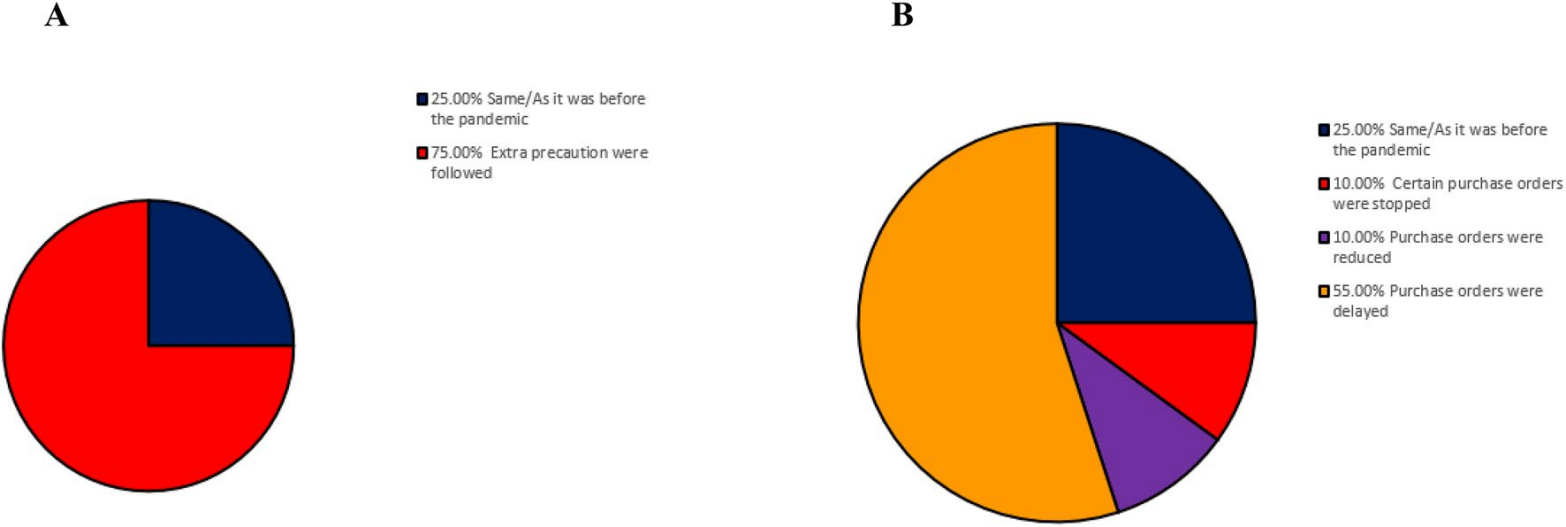
Radiopharmaceuticals preparation and purchase. (**A**) radiopharmaceuticals preparation (**B**) radiopharmaceuticals purchase.

Radiopharmaceutical preparation was prepared with more care than before pandemic; 75% (108/145) of participants prepared radiopharmaceuticals with greater care than they did before the pandemic (Fig. 3A). The purchase of materials and radiopharmaceuticals were significantly reduced *(p* < 0.001), in comparison to the normal purchase orders. Purchase was reduced to more than half the normal situation before the pandemic (64%; 64/145). According to 25% respondents, purchase was not affected and remained same as it was before pandemic; however, 5% and 6% reported that processing of some purchase orders was halted and/or put on hold respectively (Fig. 3B).

#### Scheduling

The procedure for scheduling work in the radiopharmacy lab was decided to be simplified. As a result, 45% (65/145) of all respondents have simplified their appointment scheduling procedures. However, 40% (58/145) respondents claimed that scheduling procedures remained unchanged since the pandemic, while 15% (21/145) claimed that the use of certain radiopharmaceuticals was no longer permitted.

#### Most used radiopharmaceutical

**Figure 4:**
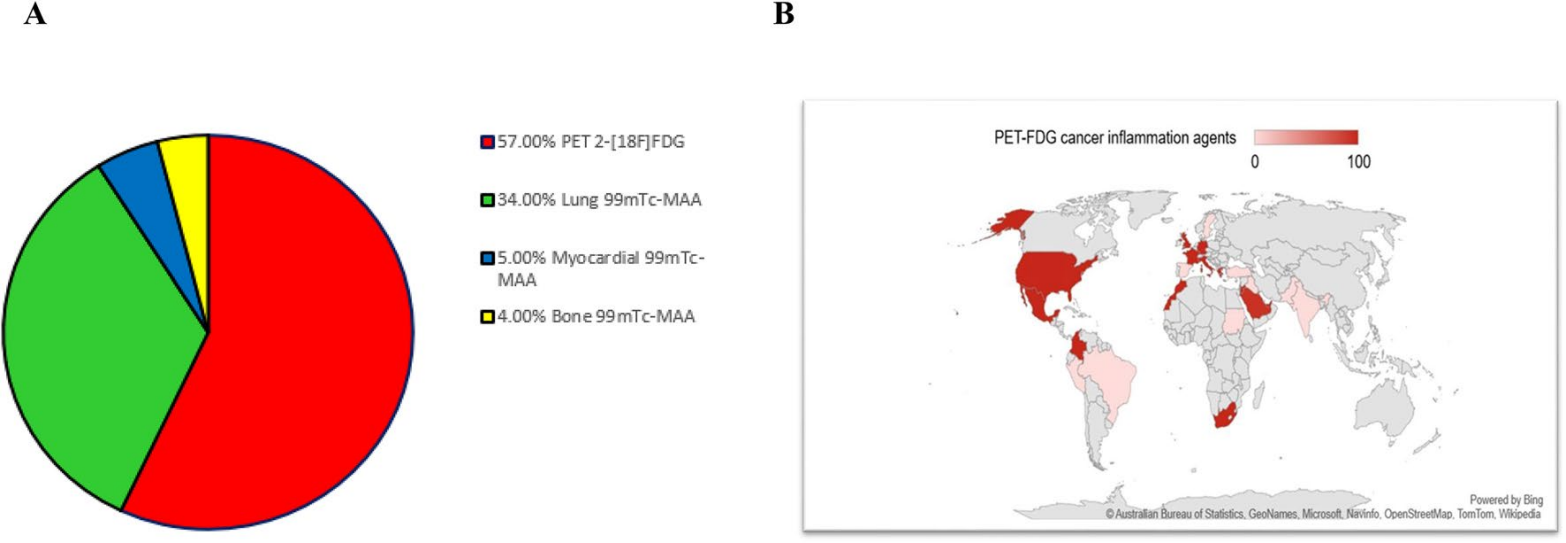
The most common imaging radiopharmaceutical used during COVID–19. (**A**) most used radiopharmaceuticals (**B**) 2-[^18^F]FDG among participants worldwide.

This survey showed that the Positron Emission Tomography (PET) radiopharmaceutical 2-deoxy-2-[18F]fluoro-D-glucose (2-[^18^F]FDG) was the most common (57%, 83/145) imaging radiopharmaceutical used during COVID–19, followed by respiratory (lung) ^99m^Tc-labeled macro aggregated albumin (^99m^Tc-MAA) of 34% (49/145). Then, the use of the cardiovascular (myocardial) systems (5%; 7/145) kits was reported followed using the musculoskeletal (bone) system (4%; 6/145) (Fig. 4). Although only a small percentage of respondents mentioned renal agents, the difference was not statistically significant.

#### Staffing levels in the radiopharmacy

The staffing level was reported in this survey. There was a 97% reduction (140/145) in staffing during the pandemic. All sections and professions almost stopped to recruit staff during COVID-19.

### Compiling data about Covid-19

70% (101/145) of participants consulted local departmental regulations while collecting measures and learning about COVID-19, compared to 30% who used social media.

## Discussion

According to the findings, the vast majority of respondents worked in the radiopharmacy of the Nuclear Medicine Department. Technologists, who are considered members of the medical profession, comprised the majority of respondents to this survey. Technologists were present and actively involved in providing services to the nuclear medicine department and radiopharmacy operations. Most of the respondents had an experience of more than 6 years in nuclear medicine and radiopharmacy activities. The pandemic has already affected the process of preparing radiopharmaceuticals, so all nuclear departments have instructed their radiopharmacy lab employees to take additional precautions when preparing radiopharmaceuticals.

The results showed the interesting observation that 2-[^18^F]FDG was reported to have been more widely used than normal during the pandemic. COVID–19 exemplifies a well-known principle underlying the use of 2-[^18^F]FDG in oncology and during inflammatory processes, such as infections. A high uptake on an image is correlated with a high number of viable cancerous or inflammatory cells, an increased glucose metabolism, high levels of GLUT transporter expression, and the activity levels of various glycolytic enzymes or metabolic activity.

Numerous studies have demonstrated that 2-[^18^F]FDG is effective for diagnosis, elucidating acute pulmonary-extrapulmonary manifestations, tracking treatment efficacy, recognising disease at an early stage, and directing medical management. These studies supported previous findings that 2-[^18^F]FDG was effective in all of these areas^11,12^ and concurred with studies that emphasised the importance of 2-[^18^F]FDG during COVID–19^13–19^.

People exposed to the SARS-CoV-2 developed respiratory complications, a healthy respiratory system is required for healthy life and to investigate the COVID-19-related complications, the respiratory ^99m^Tc-MAA represented the second order of radiopharmaceuticals widely used during COVID–19. One of the most important diagnostic procedures that nuclear medicine can perform is an imaging test that uses ^99m^Tc-MAA radiopharmaceutical. Findings of several studies supported the observation that the frequency of this usage increased during pandemic^20,21^.

The myocardial and bone systems radiopharmaceuticals were performed almost equally during the pandemic due to the importance to exclude the causes of myocardial or skeletal. According to our results and other studies’ findings, more than 28% of COVID-19-infected patients exhibit signs of cardiac injury. This is associated with a poor prognosis, a sore throat, a newly developed loss of taste or smell, fever, chills, bone and muscle pain, and other symptoms^22–24^.

Although radiopharmacy has many factors, including location, funding, financial and technical factors, and departmental conditions, 2-[^18^F]FDG and ^99m^Tc-MAA radiopharmaceuticals were the most prominent used during COVID–19.

The extraordinary presence of radiopharmaceuticals such as 2-[^18^F]FDG and ^99m^Tc-MAA at COVID-19 was unaffected by geographical location, radiopharmaceutical production sources, or radiopharmaceutical production types. There was no variation in how individuals responded to the virus. Our survey proved the importance of these radiopharmaceuticals. We will expand on this knowledge to enhance the research that has already been conducted on the application of radiopharmaceuticals, and we will prioritise new features.

The process of scheduling was revealed to be reduced during the pandemic, this added support to the lack of staff and the reduced staffing levels. In addition, the use of certain radiopharmaceuticals was discontinued during the pandemic, which further supports the reduced pattern of purchasing. Similarly, Giammarile et al.^25^, also reported a significant reduction in nuclear medicine diagnostic and therapeutic procedures in June 2020 (73%) and October 2020 (56.9%) due to pandemic-related changes. The study also found that out of different nuclear medicine procedures, oncological PET tests, exhibited lower reduction in utilisation than the conventional nuclear medicine, especially nuclear cardiology. Moreover, in high-income nations, the detrimental effect was less evident. Gradually the situation of the supply chains of radioisotopes, generators and other essential materials is improving and showing the trend same as pre-Covid-19 time^25^. A European study reported that most of the European countries included in the study did not face any issue with the supply of radiopharmaceuticals^26^.

In the radiopharmacy lab, there was no standardised schedule to follow for the preparation of radiopharmaceuticals or specific uses. As reported by Moreira et al.^26^ in most European nuclear medicine departments the most common organizational changes were alteration of scheduling practices, like rotating cohort teams of personnel to avoid widespread quarantine^26^. Additionally, the production schedule was modified to account for decreased demand resulting from staff reductions, fewer purchases, transportation issues, and additional hygienic precautions and regulations. COVID-19 impacted purchase orders, supply chains, how individuals work, and what they purchase. Numerous pandemics have been suggested to be cured by virtual reality^27–30^.

The regional lockdown, increased border controls, cancellation of most commercial passenger flights, and increases in cargo costs have reduced, delayed, or halted the availability and supply of vital medical radioisotopes. These radioisotopes are used in a variety of applications, including radiopharmacy, nuclear medicine, and research. This is true in light of the findings, which indicate that the supply and availability of these radioisotopes have been reduced, postponed, or completely halted^25,31–35^.

A significant proportion of respondents followed the regional department's rules. More people were gathering information about COVID-19 via various social media platforms. This is similar to the findings of multiple studies that cited World Health Organization, National Energy Commission, and International Atomic Energy Agency^36–39^. According to the findings of this study, the rules regarding this pandemic need to be updated in order to remain inside the existing local regulations^40,41^. The guidelines for the clinical practise of nuclear medicine and radiopharmacy should be made available as soon as possible during COVID-19. This action will benefit the management of future COVID-19 variants such as late Delta and Omicron^42–44^.

We determined that the most efficient way for us to accomplish our goals here would be to conduct a simple local survey. We searched for and located basic data or databases that we could use to initiate this project. We discovered few relevant studies and surveys on COVID-19 for the radiopharmacy lab work of the nuclear medicine department. This created a challenge for us because we required more information on the subject. We have presented evidence in this survey that the nuclear medicine industry, lacks some kind of standardisation, particularly with regard to the surveys presented here. When we talk about standardisation, we're talking about a set of agreements or procedures that ensures a method or piece of work meets a standard. All relevant groups, divisions, service providers, and organisations in an industrial society must adhere to predetermined quality, agreement, uniformity, and comparability guidelines. This guarantees the success of design, production, and service.

The COVID-19 pandemic has had a significant negative impact on nuclear medicine therapeutics, clinical/imaging settings and research activities. Despite this significant impact, the researchers are embracing an attitude that “the show must go on”. At the global level, the continuation of vital nuclear medicine services has been given priority. In the pandemic time, education on nuclear medicine was adapted. The research activity on nuclear medicine was not stopped during pandemic, rather the research activities continued significantly leading to emerging indications, innovative radiopharmaceuticals, and new imaging/data analysis techniques^45^.

## Conclusions

According to the findings of this survey, COVID-19 made it difficult to conduct radiopharmacy business, duties, and activities, which hampered nuclear medicine research. The outstanding presence of 2-[^18^F]FDG and ^99m^Tc-MAA radiopharmaceuticals during COVID-19 provide an opportunity and a scientific priority to study and research. There is a need for creative, quantitatively sound, and context-appropriate action plans. During and after the pandemic, standard regulations, instructions, patient care, recommendations, and protection should be implemented. There is currently a shortage of personnel in the highly specialised fields of nuclear medicine staffing and radiopharmacy, which may be responsible for at least some of the challenges. The number of people involved in radiopharmacy activities is expected to increase in the future. As a result, both the existence of a reliable information database and the standardisation of this process will be possible to avoid any other hurdles in the nuclear medicine and radiopharmaceuticals.

## Data Availability

The data are available within the paper.
